# Ocular-Surface Regeneration Therapies for Eye Disorders: The State of the Art

**DOI:** 10.3390/biotech12020048

**Published:** 2023-06-15

**Authors:** Matteo Posarelli, Davide Romano, Davide Tucci, Giuseppe Giannaccare, Vincenzo Scorcia, Andrea Taloni, Luca Pagano, Alfredo Borgia

**Affiliations:** 1 St. Paul’s Eye Unit, Department of Corneal Diseases, Royal Liverpool University Hospital, Liverpool L7 8YE, UK; mposarelli@gmail.com (M.P.); luca.pagano91@hotmail.it (L.P.); alfr.borgia@gmail.com (A.B.); 2 Ophthalmology Unit, Department of Medicine, Surgery and Neuroscience, University of Siena, 53100 Siena, Italy; 3 Eye Clinic, Department of Neurological and Vision Sciences, University of Brescia, 25123 Brescia, Italy; davide.romano.md@gmail.com; 4 Eye Unit, University Hospitals of Leicester, NHS Trust, Leicester LE1 5WW, UK; 5 Department of Biomedical and Surgical Sciences, Section of Ophthalmology, S. Maria Della Misericordia Hospital, University of Perugia, 06123 Perugia, Italy; davidetucci@live.it; 6 Department of Ophthalmology, University Magna Græcia of Catanzaro, 88100 Catanzaro, Italy; vscorcia@unicz.it (V.S.); taloni.oculistica@gmail.com (A.T.); 7 Eye Unit, Humanitas-Gradenigo Hospital, 10153 Turin, Italy

**Keywords:** ocular-surface disease, limbal stem cells, tissue regeneration, dry-eye disease, neurotrophic keratopathy, autologous serum tear, amniotic membrane

## Abstract

The ocular surface is a complex structure that includes cornea, conjunctiva, limbus, and tear film, and is critical for maintaining visual function. When the ocular-surface integrity is altered by a disease, conventional therapies usually rely on topical drops or tissue replacement with more invasive procedures, such as corneal transplants. However, in the last years, regeneration therapies have emerged as a promising approach to repair the damaged ocular surface by stimulating cell proliferation and restoring the eye homeostasis and function. This article reviews the different strategies employed in ocular-surface regeneration, including cell-based therapies, growth-factor-based therapies, and tissue-engineering approaches. Dry eye and neurotrophic keratopathy diseases can be treated with nerve-growth factors to stimulate the limbal stem-cell proliferation and the corneal nerve regeneration, whereas conjunctival autograft or amniotic membrane are used in subjects with corneal limbus dysfunction, such as limbal stem-cell deficiency or pterygium. Further, new therapies are available for patients with corneal endothelium diseases to promote the expansion and migration of cells without the need of corneal keratoplasty. Finally, gene therapy is a promising new frontier of regeneration medicine that can modify the gene expression and, potentially, restore the corneal transparency by reducing fibrosis and neovascularization, as well as by stimulating stem-cell proliferation and tissue regeneration.

## 1. Introduction

**Figure 1:**
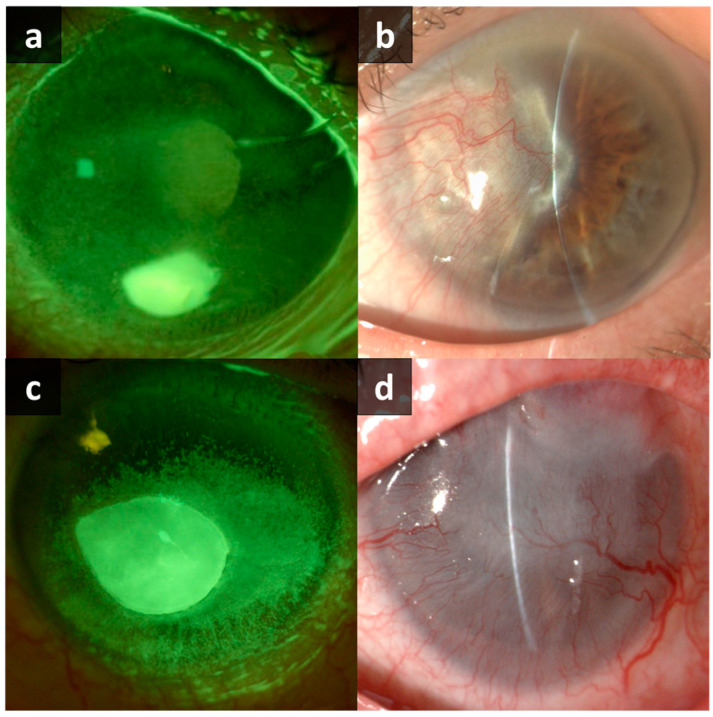
(**a**) Dry-eye keratopathy in a patient with Sjögren’s syndrome; (**b**) advanced pterygium encroaching on the visual axis; (**c**) neurotrophic corneal ulcer associated with diffuse corneal fluorescein staining; and (**d**) full corneal conjunctivalization in limbal stem-cell deficiency six months after a chemical injury.

The ocular surface is a complex structure that includes the cornea, the conjunctiva, the main and accessory lacrimal glands, the meibomian glands, and the eyelashes. All these components are strictly connected by the epithelia, the nerves, and the immune, vascular, and endocrine systems [1]. The eye is constantly exposed to evaporative stresses, but the ocular-surface structures work together to maintain cornea integrity through homeostatic mechanisms. When one of these mechanisms is altered, there is a loss of ocular-surface homeostasis, and, subsequently, corneal damage (Figure 1) [2].

When the eye surface is altered by a disease, the damage to cornea structures without self-renewal capacity, such as the limbus, the stroma, or the endothelium, can lead to corneal opacification and visual acuity impairment. Although standard treatments, such as corneal transplants, aim to restore the corneal transparency, the shortage in corneal donors, the risk of rejection, and the limits of graft survival are critical problems that raise the need for strategies to promote tissue regeneration.

Regenerative therapy offers a distinct advantage over traditional methods in treating ocular-surface diseases. Rather than simply managing symptoms, regenerative therapy focuses on restoring damaged ocular surfaces by promoting the regeneration of corneal tissues and improving tear film stability, providing long-term relief, improving visual outcomes, and significantly enhancing the quality of life for patients with ocular-surface diseases. Additionally, this new approach may help in improving clinical outcomes, reducing the need for corneal tissues, and allowing clinicians to treat a larger number of patients. In the field of tissue engineering, biomaterials with controlled-release capabilities, such as multilayered hydrogels and scaffolds, have advanced significantly [3]. Disease understanding is pivotal for successful polymer-based biomaterials. However, selecting the right polymer remains challenging, as interaction with host immune cells can impact outcomes negatively. Researchers and physicians are dedicated to enhancing biomaterials to ensure long-term tolerance and optimal medical performance. Complex implant materials must adhere to strict quality standards to prioritize patient safety. Biomaterials necessitate qualities such as strength, resistance, elasticity, hardness, density, and biocompatibility to fulfill their intended purpose effectively [4]. In a recently published paper, Kumar et al. reviewed up-to-date options for corneal stromal, limbal, and nerve regeneration with special emphasis on stem-cell secretomes and exosomes [5]. In another comprehensive review, Amador et al. discussed the gene-delivery system and editing technique, and epigenetic treatments [6]. Viral vectors, as well as nanoparticles and nanopolymers, are minimally invasive approaches that are promising results in in vitro or animal models studies. Further, El Zarif et al. reviewed the most recent therapies for corneal stromal regeneration for advanced keratoconus with particular focus on autologous adipose-derived adult stem cells (ADASCs), decellularized human corneal stroma, allogenic lenticule, and corneal inlay [7]. 

Herein, we review the different strategies employed in ocular-surface regeneration (Table 1), including cell-based therapies, growth factor-based therapies, and tissue-engineering approaches. Although many anterior segment diseases affect the cornea, in this paper we included all the different components of the ocular surface, such as the corneal layers, the limbus, the conjunctiva, and the lacrimal apparatus.

## 2. Corneal Ocular Surface 

### 2.1. Dry-Eye Disease

Dry-eye disease (DED) is a multifactorial ocular condition characterized by instability and loss of homeostasis of the tear film, ocular inflammation and damage, and neurosensory abnormalities [8]. According to the Tear Film and Ocular Surface Society’s Dry Eye Workshop II (TFOS DEWS II), it is usually classified as aqueous deficiency, in which there is a dysfunction of the lacrimal glands—evaporative, in which the most common cause is Meibomian gland dysfunction (MGD), and mixed forms. Although the availability of various artificial tear substitutes to maintain the tear film integrity, many patients develop severe ocular-surface damage [9,10]. Further, it is demonstrated that a persistent inflammation can alter the corneal nerve function leading to a vicious cycle characterized by altered tear production, neurosensory abnormalities, severe corneal damage, and impaired corneal sensation (Figure 1a) [11]. In this scenario, the most recent treatments aim to reestablish the ocular-surface integrity by restoring the corneal biological components such as nerve-growth factors (NGF), epithelial-growth factors (EGF), nutrients, and vitamins [8]. Autologous serum tear (AST) is a blood derivate used topically for patients with severe DED. Initially introduced in 1970 to treat subjects with severe ocular-surface damage caused by Stevens–Johnson syndrome, chemical burns, or Sjögren syndrome, it has more recently gained popularity for less severe conditions, such as DED. It contains EGF, NGF, insulin-like growth factor (IGF-1), vitamins, and fibronectin in concentrations similar to those observed in the human tears, and in vitro and in vivo studies have demonstrated these components can enhance the corneal epithelial healing process [12]. Although cell-culture studies reveal an increased epithelial proliferation with 20% diluted concentrations of AST, epithelial migration seems more efficient with higher concentrations ranging between 50% and 100% [13,14]. In a randomized controlled trial, Urzua et al. observed a 50% improvement in OSDI score in patients with severe DED using 20% AST [15]. Further, other authors have used higher AST concentrations of 60–80% and have shown an improvement in corneal TBUT and fluorescein score in randomized trials [16,17,18]. An allogenic preparation of the AST could represent a valid option in these subjects, however, there are some evidences that an allogenic serum could stimulate an immune cross-reaction between the host receptors and the donor antigens [9]. The umbilical-cord serum has a higher concentration of EGF and NGF than AST, and it can be safely used in patients with systemic diseases [19]. Alio et al. conducted a prospective study in 18 subjects with severe DED and observed an improvement in symptoms and corneal staining of 89% and 72%, respectively, after one month of use of umbilical-cord serum [20]. Recombinant human nerve growth factor (rh-NGF) has demonstrated its efficacy in regulating the growth, proliferation, and survival of neurons. Although it is mostly used in patients with neurotrophic keratopathy (NK), studies have shown that rh-NGF can restore the corneal sensation and tear production in in vivo models of DED [21]. 

The preliminary results from a four-week phase III, multicenter, double-masked, vehicle-controlled clinical trial suggest that the use of Ngf in individuals with Sjogren’s syndrome is associated with a significant improvement in the Schirmer test after the four-week treatment period [22].

In recent years, ocular-surface biomarkers have been studied for their potential role in dry-eye disease pathogenesis and treatment [23]. Among the identified molecules, proinflammatory cytokines, such as interleukin-6 (IL-6), tumor necrosis factor alpha (TNF-α), and interferon gamma (IFN-γ), have been studied for their implications in the release of acute-phase proteins and metalloproteinases (MMP) [24,25,26]. In addition, MMP-9 molecules have also been implicated in leucocytes migration and worsening of ocular-surface inflammation [27,28]. These biomarkers are considered important for assessing patients with DED and staging the severity of the inflammation, and future therapies could aim to target these molecules to limit the inflammatory burden and, potentially, stimulate the corneal repair processes.

### 2.2. Neurotrophic Keratopathy

**Figure 2:**
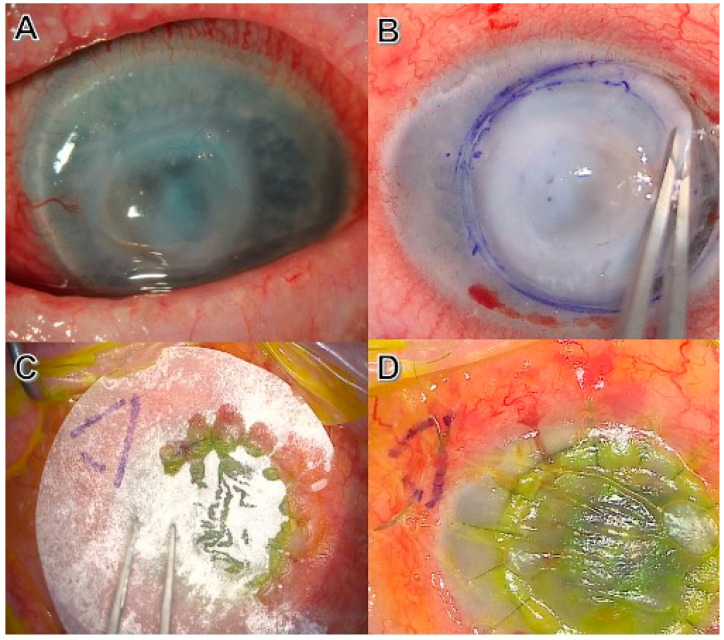
(**A**) Corneal perforation in an advanced neurotrophic ulcer; (**B**) corneal debulking during the early phases of surgical management: penetrating keratoplasty + open sky cataract extraction + amniotic membrane transplant (Omnigen^®^); (**C**) dry amniotic membrane (Omnigen^®^) apposition on the completed penetrating keratoplasty; and (**D**) amniotic membrane sutured on the ocular surface as a graft using a 10/0 absorbable (Vicryl) continuous suture.

Neurotrophic keratopathy (NK) is a severe corneal disease characterized by a loss in corneal sensation and impaired corneal nerve function (Figure 1c) [29]. When the nerves are damaged, the reduction in corneal sensation and trophic factors can lead to a breakdown of the corneal epithelium, and, subsequently, of the ocular-surface integrity and function [30]. The NK management is complex and aims to maintain the epithelial integrity, reduce the epithelium breakdown, and restore the ocular-surface integrity. Besides the classic therapies to lubricate the cornea, new treatments have been recently introduced to restore the corneal nerve function, the corneal sensation, and the lacrimal function. The recombinant human nerve growth factor (rhNGF) is a molecule biochemically identical to the human growth factor and has been approved in 2017 by the European Medical Agency for use in patients with neurotrophic keratopathy. This treatment represents a promising new drug because in vitro and in vivo studies have demonstrated its capability to restore the nerve integrity and corneal sensation by binding the NGF receptors P75NTR and TrkA [31]. In a randomized, controlled, double-blinded trial, 156 patients with stage 2 NK were enrolled to receive rhNGF or vehicle [32]. The subjects were treated for eight weeks, and the authors observed a significant improvement or complete epithelial healing in the NGF group compared to the vehicle group (74% vs. 43.1%, respectively). In a prospective, interventional study, Mastropasqua et al. used in vivo confocal microscopy (IVCM) to assess the corneal nerve density in patients with NK stages 2 and 3 [33]. Their results showed a significant improvement in corneal nerve density and corneal sensation after eight weeks of treatment with rhNGF. The amniotic membrane is the inner, avascular layer of the fetal membrane, and it is widely used in ophthalmology for its anti-inflammatory, regenerative, and anti-scarring properties [34]. It is characterized by three layers: a monolayer epithelium, a thick basement membrane similar to the human membrane, and an avascular stroma. It contains various molecules that stimulate the healing of the epithelium, such as NGF, keratinocyte growth factor, and hepatocyte growth factor, and the thick basement membrane serves as scaffold for the limbal migration of epithelial cells on the corneal defect [34]. Although clinical trials have not shown a clear beneficial effect, the use of amniotic membrane in patients with NK can help in healing the epithelial defect. If the membrane is incorporated in the cornea and the epithelium migrates over, the amnion is defined as a “graft” (Figure 2), whereas it is called a “patch” if the epithelium moves under and the membrane falls off [29]. 

The basement membrane component of the amniotic membrane enhances support for epithelial cells, limbal stem cells, and corneal transient amplifying cells, as it shares a similar composition to the conjunctiva [35]. It also maintains clonogenicity, which encourages the differentiation of both goblet and non-goblet cells while preventing the presence of inflammatory cells and their protease activities [36]. Furthermore, the stromal side of the amniotic membrane hinders the transformation of normal fibroblasts into myofibroblasts, thereby reducing the formation of scars and blood vessels [36].

Further, in the case of stromal lysis or melting, the amnion can promote keratocytes migration into the cornea, a process that can reduce tissue scarring. Today, new devices are available that combine contact lenses with amniotic membranes, such as OmniLenz^®^ and ProkeraTM, and allow clinicians to apply the membrane without the need of sutures [34]. In selected cases, a conjunctival flap can be used to promote corneal healing and reduce the risk of corneal perforation. The bulbar conjunctiva is transplanted on the cornea surface to replace the unhealthy stroma, provide basal tissue for the growing epithelium, blood supply, and growth factors [29]. Corneal neurotization is a difficult surgical technique that allows the restoration of the corneal sensation using branches of the trigeminal nerve of the contralateral eye [37]. The procedure maintains the anatomical integrity of the eye, but it needs to be used by expert surgeons.

### 2.3. Neuropathic Corneal Pain

Neuropathic corneal pain is defined as a pain caused by an alteration or dysfunction of the nervous system [38]. Tissue damage and inflammatory processes can result in increased responses from peripheral nociceptors with intensification of the pain signal. This mechanism alters the physiological body response to pain and, over time, can result in receptor sensitization, centralization of the pain, and, in severe cases, spontaneous pain perception [39]. Neuropathic corneal pain (NCP) can be caused by peripheral nerve injuries and systemic diseases, and the diagnosis and management of the disease require skilled ophthalmologists with experience in the field. Since in many patients IVCM studies have shown nerve alterations and the presence of microneuromas, treatments aim to promote neuroregenerative processes to cure the underlying etiology that caused the abnormal pain perception [39]. AST contains neurotrophic factors, IGF-1 and NGF, and various studies on DED patients have shown improvement in ocular symptoms [40]. In NCP, Hamrah et al. have demonstrated an improvement in photoallodynia and allodynia with 20% AST, and a significant increase in corneal nerve density [18,39,41]. The amniotic membrane is used in NCP patients because it can alleviate symptoms in the case of peripheral pain. However, especially with Prokera^®^ amniotic membrane, some subjects are not able to tolerate the polycarbonate ring. In this case, it is possible to remove the ring and place the membrane directly into a bandage contact lens [39].

## 3. Corneal Limbus

### 3.1. Pterygium

Pterygium is an abnormal subconjunctival fibrovascular growth that encroaches on the cornea, causing clinically significant irregular astigmatism proportioned to the amount of visual axis involved (Figure 1b) [42,43]. It is usually bilateral, affecting the nasal side of the conjunctiva and cornea. Pathogenesis is still nowadays not fully discovered; however, it has been hypothesized that a major role is due to sunlight exposure [42]. UV lights may damage corneal limbus, with a localized deficit of limbal stem cell, and subsequent activation of growth factors which results in angiogenesis and cell proliferation. A deficit of limbal stem cell causes corneal conjunctivalization [42]. Pterygium can be primary, if the patient has no history of previous surgical management, or recurrent, in the case of its regrowth after previous surgery, due to reactivation of the inflammation process and is highly associated with the pterygium surgical removal technique chosen [44]. Diagnosis is primary clinical at slit lamp and the following aspects must be evaluated: size, location, degree of vascularization, extent, and degree of corneal involvement. In pterygium, it is possible to notice three different parts: a body (which is the core of pterygium, with wing shape localized within the bulbar conjunctiva), a cap (leading edge of pterygium on the cornea, avascular, and rich in fibroblast) and head (highly vascularized, localized behind the cap, and firmly attached to the cornea) [43]. Differential diagnosis must be conducted with ocular-surface squamous neoplasia and skin cancer [45]. Management of pterygium is surgical, and a wide range of techniques have been described: bare sclera, conjunctival flap, conjunctival autograft, limbal conjunctival autograft, and amniotic membrane. Adjuvant therapies for pterygium management have also been used, such as mitomycin C, beta-radiation, 5-fluorouracil (5-FU), topical use of bevacizumab, and interferons [46]. The goal of surgery is to excise the pterygium, restore the cornea surface, and prevent the risk of pterygium recurrence.

“Base sclera technique” is one of the simplest surgical techniques and involves removing the pterygium and performing a tenonectomy to expose the underlying sclera. However, this approach is associated with a high recurrence rate, which can reach up to 88% [47]. Additionally, leaving sclera unprotected increase the risk of scleral melting, formation of pyogenic granuloma, and delayed reepithelization of the cornea [48]. 

The conjunctival autologous graft (CAG) consists in covering the bare sclera with a free graft of autologous conjunctiva after the pterygium removal, and it is nowadays the preferred approach given lower incidence of recurrence, usually between 3.3% and 16.7% in the case of primary pterygium [49], and around 33% in the case of treatment of pterygium recurrence [50]. The free autograft can be fixated with sutures (commonly Vicryl 8/0), fibrin glue, or the patient’s autologous blood [46,51]. No significant differences in pterygium recurrence among these three techniques are reported in the literature according to meta-analysis, however, the conjunctival autograft with fibrin glue presents a lower risk of graft displacement and conjunctival retraction, and also a shorter surgery time, compared to the other two techniques [52,53]. An alternative to conjunctival autograft is the use of an amniotic membrane (AM) graft, in which the bare sclera is covered by a patch of amniotic membrane graft, which, similar to CAG, can be sutured or glued. Compared to CAG, AM has a higher risk of pterygium recurrence (6.7–40.9%), but lower compared to bare sclera [46,49,54]. Several adjuvant treatments have been evaluated in the literature to reduce the risk of pterygium recurrence after surgical removal following all the aforementioned techniques. These can include antimetabolites, such as mitomycin C (MMC) and 5-fluorouracile (5-FU), anti-vascular endothelial growth factor (bevacizumab) as cyclosporin, and the use of irradiation [55,56,57]. According to a meta-analysis by Zeng et al., the use of MMC has the lowest risk of pterygium recurrence, in both bare sclera and CAG techniques, followed by beta-radiotherapy and bevacizumab [56]. The use of 5-FU was not superior to a placebo. The use of cyclosporin A can reduce the risk of recurrence only in the case of the bare sclera technique and not in the case of CAG [58]. Considering the ratio risks/benefits among all the adjuvant treatments, the best option would be the use of 0.2 mg/mL of MMC applied for 3 min with conjunctival autograft. This combination in a study by Frucht-Pery et al., granted 0% of pterygium recurrence, versus 6.6% of bare sclera + MMC, and 46.6% of CAG without MMC [59]. Looking ahead, the focus in pterygium research is on identifying genetic and molecular factors that contribute to the risk of recurrence, with the goal of developing medical therapies. Research has found abnormal expression of platelet-derived growth factor receptor β (PDGFR-β) in the stroma of pterygium, suggesting that its inhibitor, sunitinib, could, potentially, be used as a treatment [60].

### 3.2. Limbal Stem-Cell Deficiency

Corneal limbus is an area of approximately 2 mm of diameter, with ring shape, placed between the cornea and the bulbar conjunctiva and is the niche of limbal stem cells (LSCs), which are adult stem cells that differentiate into corneal epithelial cells [61,62,63].

**Figure 3:**
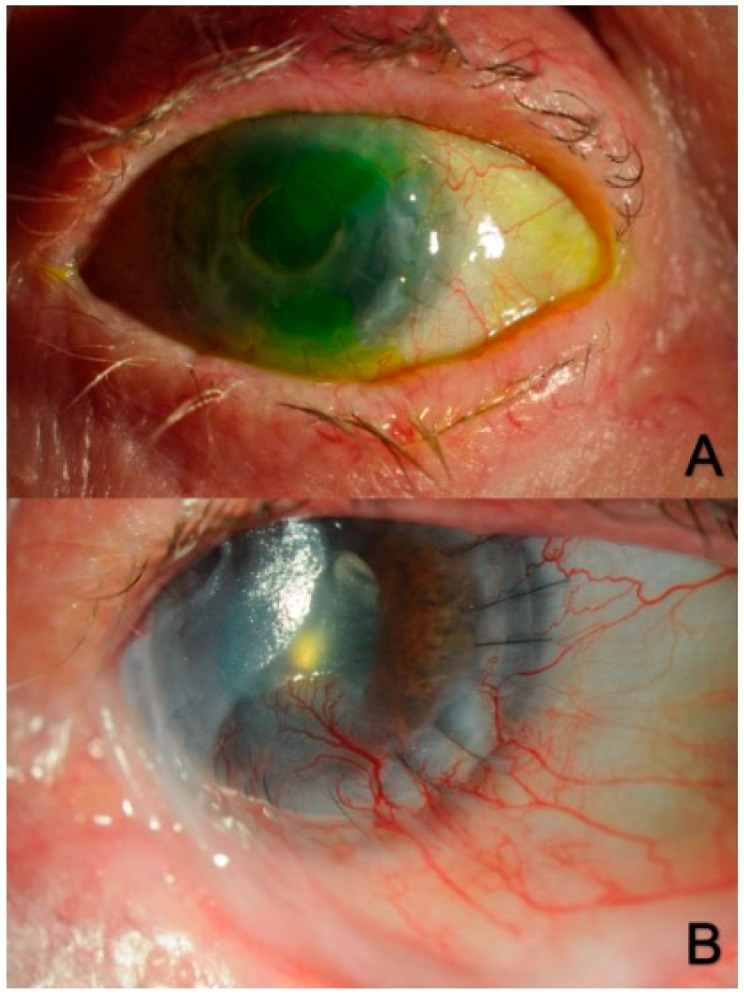
Limbal stem cell deficiency (LSCD) is associated with corneal epithelial abnormalities (**A**), corneal conjunctivalization and neovascularization (**B**), corneal scarring, and chronic inflammation.

**Figure 4:**
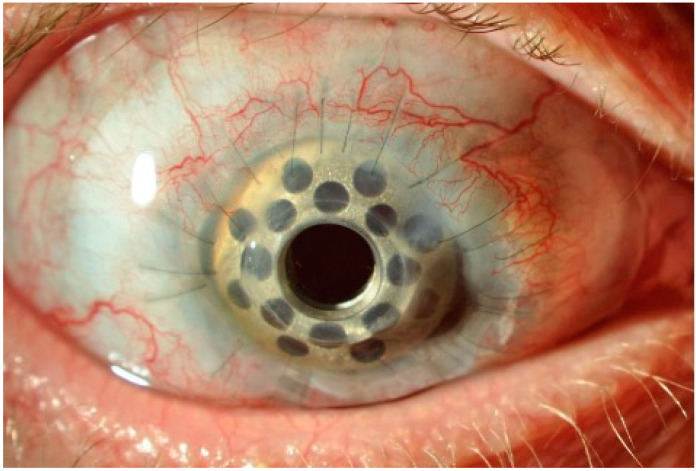
The Boston keratoprosthesis (KPro) Type 1 in a patient with bilateral total limbal stem-cell deficiency (LSCD).

The integrity of limbus is essential for the physiological turnover of LSCs and, subsequently, for corneal epithelial cells, and the related transparency of the cornea [61,62]. Additionally, LSCs act as a barrier to prevent the conjunctival epithelial cells from migrating over the corneal surface, causing the conjunctivalization of the cornea (Figure 1d) [61,62]. Therefore, limbal stem-cells deficiency (LSCD) results in poor epithelization of the cornea, inflammation, vascularization, and scarring (Figure 3) [64,65]. Causes of LSCD can be congenital, traumatic, autoimmune, and idiopathic [65]. The diagnosis is primary clinical, with loss of Vogt palisaded, progressive conjunctivalization, and superficial neovascularization of the cornea, and instability of the corneal epithelium [64,65]. The management of the disease is challenging and usually requires surgical treatments, especially when there is an involvement of the visual axis [65]. Nowadays, there are multiple techniques for limbal stem-cell transplantation (LSCT), which can be divided accordingly between the anatomic graft transplanted and the donor (allogenic or autologous) [5,66,67,68,69,70,71]. In the case of allogenic LSCT, the donor can be cadaveric or a living relative [66,72]. Based on the type of anatomic graft, there are conjunctival limbal graft (autologous: CLAu; allogenic: CLAL), allogenic keratolimbal graft (KLAL), and limbal epithelial transplantation [72]. If the LSCD affects only one eye, autologous transplants are recommended, whereas in the case of bilateral involvement, allogenic LSCTs are needed [65]. The choice of which type of LSCT to perform depends on the grade of involvement of the ocular adnexa and the bilaterality of the disease [65]. In the case of LSCD due to cicatrizing ocular-surface disease with symblepharon, conjunctival limbal graft (CLAu in case of monoliteral involvement, or CLAL in case of bilateral involvement) should be preferred, whereas in the case of no adnexa involvement, the other LSCT procedures are a viable option. KLAL involves the transplantation from a cadaveric donor of two corneo–scleral rims to restore at 360° the host limbal tissue [73]. Cultivated limbal epithelial transplantation (CLET) requires a small biopsy of 2 mm × 2 mm of limbus from which the epithelial limbal stem cells are harvested and cultivated in vitro, obtaining a sheet of limbal epithelial stem cell, which is, subsequently, transplanted onto the affected eye. Cultivation can be performed accordingly in two methods: suspension or explant. The first requires that harvested cells are enzymatically treated and seeded on amniotic membrane, a fibrin carrier, or a plastic culture dish covered by fibroblast [74,75]. In the second method, instead, the harvested cells are scaffolded on de-epithelized amniotic membrane with subsequent stem cell expansion in three weeks [76,77]. CLET technique requires the use of autologous or allogenic limbal epithelial cells which are cultivated in vitro and then transplanted onto the cornea [72]. The simple limbal epithelial transplantation (SLET) differs from CLET in the fact that the epithelial cells are directly transplanted onto the corneal surface of the host using an amniotic membrane and fibrin glue or a dual layer of amniotic membrane harvested limbal stem cell in between [78,79]. The expansion of epithelial limbal stem cells is, therefore, not obtained in laboratory cultivation, but in vivo on the ocular surface of the affected eye. Cultivated oral mucosa epithelium (COMET) requires an autologous biopsy of labial of buccal mucosa, from which the epithelial cells are then cultivated, usually on the amniotic membrane and transplanted onto the host cornea [80,81,82]. This technique has the advantage to not need immunosuppression, however, its outcomes are less favorable than LSCT because of a higher risk of persistent epithelial defects, cornea neovascularization, and rejection [65]. Keratoprosthesis is, typically, considered as a last resort in cases of bilateral total limbal stem-cell deficiency (LSCD) with extensive ocular adnexal involvement, significant symblepharon, and previous failed limbal stem-cell transplantation (LSCT) [83]. Multiple types of keratoprosthesis (KPro) are available and the choice can be reached according to the presence or not of aqueous deficiency dry eye (ADDE): Boston KPro type 1 (Figure 4) or Aurolab KPro (auroKPro) in the case of absence ADDE, or Boston KPro type 2, LV Prasad KPro (LVP KPro), or modified osteo-odontokeratoprosthesis (MOOKP) in the case of presence of ADDE [65]. The KPro types have a complex follow-up management, steep learning curve, and high risk of post-operative complications, such as endophthalmitis, retroprosthetic membrane formation, glaucoma, and retinal detachment [84,85,86]. The lack of comparative systematic review and meta-analysis prevent the proper addressing of the success rate of one type of KPro over the other in the case of LSCD. Currently, future prospective management of LSCD mainly focuses on the use of cell-based therapies [87,88]. These include adipose tissue-derived mesenchymal stem cells, embryonic stem cells, and induced pluripotent stem cells [83,88], which have corneal wound-healing, scarring-remodeling, and angiogenesis properties [65].

## 4. Corneal Endothelium

### Fuchs Dystrophy

Fuchs endothelial corneal dystrophy (FECD) is the most common form of corneal dystrophy, and is characterized by a reduction in human corneal endothelial cells (hCEC) at a higher rate than normal. hCEC are stuck in the G1 phase of their vital cycle and are unable to replicate, so their number decreases by a rate of approximately 0.6% per year [89].

**Figure 5:**
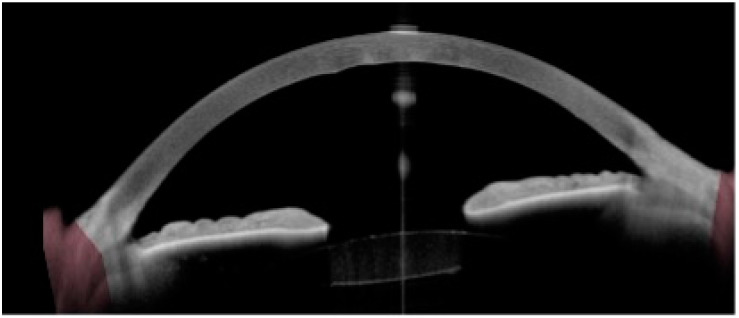
Anterior segment OCT (AS-OCT) showing an abnormal corneal morphology in Fuchs endothelial dystrophy. The corneal stroma is edematous with a central corneal thickness of 638 microns and posterior stromal ripples.

Over time, this condition can lead to endothelium decompensation, and, progressively, to corneal oedema, scarring, and, eventually, blindness (Figure 5). FECD is usually treated with endothelial keratoplasty, but its application is currently limited by an international shortage of donor tissue, with only one donor available for every 70 patients requiring transplantation [90]. In consideration of this shortage, many approaches are being tested and used to overcome the problem and treat the condition; these methods mainly aim to regenerate the endothelium without the need for any graft and can be divided into surgically based approaches and cell-based approaches [91]. The ability of corneal endothelium to heal after an iatrogenic trauma was first and accidentally noted many years ago by several authors [92,93]; different cases have been reported of patients with an inadvertent removal of DM during routinary cataract surgery, in which post-operative assessments highlighted how the endothelial cells had migrated to cover the bare stromal surface. These reports seemed to suggest that corneal endothelial cells can effectively compensate with rearrangement and migration the loss of tissue surgically removed. Further evidence supporting this hypothesis can be found in other studies in which patients undergoing posterior lamellar keratoplasty for FECD, despite a detached graft, still reached a good clearing of the cornea and a partial endothelization through migration of endothelial cells from the peripheral cornea [94,95]. In consideration of these findings, some surgeons started performing a surgical maneuver of descemetorhexis without any endothelial graft; the results were variable, mainly depending on the descemetorhexis diameter and the integrity of the DM after endothelium removal [95,96] with a high failure rate described for 8 mm stripping [97], mixed outcome reported for cases treated with 6.0–6.5 mm Descemetorhexis [98,99], and good results, finally, achieved with more limited stripping of 4 mm [100,101,102].

These observations led many surgeons to start using the novel surgical approach of Descemet stripping without endothelial grafting, alternatively termed “descemetorhexis without endothelial keratoplasty” (DWEK) or “Descemet stripping only” (DSO), and a recent meta-analysis confirmed that, despite the lack of comparative studies, this technique seems to effectively improve visual acuity and pachymetry in early stages of FECD [103]. In the attempt to promote hCEC migration in patients affected by FECD and undergoing DWEK/DSO, recently, RHO-kinase inhibitors (ROCK-I) have been used as an adjuvant to endothelial surgery [104,105]. RHO-kinase enzymes are a group of proteins involved in the modulation of structural change in the internal cell cytoskeleton, inhibition of smooth muscle, vasodilation and cellular delamination, and migration [106]. For these reasons, since their discovery, they have been subject to various research in ophthalmology as therapeutic targets, especially for conditions with low endothelial cell counts. However, although via kinase pathways proliferative effects may effectively be modulated, it is unlikely that ROCK-I will ever safely induce mitotic activity of HCEC. Indeed, they have instead been successfully used in the attempt to promote hCEC migration [107]. 

The first double-armed prospective study of ROCK-I in DWEK/DSO surgery was published a few years ago, proving that the use of ripasudil leads to a faster corneal recovery and to a higher central endothelial cell count [108], and the results from another trial that came out later highlighted similar outcomes [109]. Even if these results are encouraging, further studies are still necessary to assess which patients are suitable for the treatment. 

Currently, a Phase 1 double-masked, randomized clinical trial is assessing the safety and efficacy of a specific treatment called human corneal endothelial cell therapy (HCEC-1) in adult patients with corneal oedema due to endothelial dysfunction [110]. 

Recently, cell-based approaches have grown in popularity to overcome the worldwide shortage of eye donors. These techniques mainly rely on the capacity of in vitro expansion of isolated primary hCEC when given the appropriate stimuli [111]. These expanded cells need then to be delivered onto the inner corneal surface, where they can effectively act as intended and restore endothelial function. This delivery can be achieved through endothelial cell sheet transplantation or through cell injection into the anterior chamber. For the endothelial cell sheet transplantation, two main techniques have been proposed: primary hCEC isolated from cadaver donor corneas or differentiated stem cells and cell lines [112]. In the case of primary hCEC taken from human cadaveric donors, it has been noted that many donor factors have a significant impact on the culture success rate, such as cell density [113], cause of death, previous surgery in the eye, overall health, tissue storage time [114,115], age (with a lower proliferation capacity seen in older donors) [116], and the region of the cornea where hCEC belongs (with cells from the periphery having a higher proliferative capacity) [115,116]. In the case of stem cells and hCEC-like cells with stem-cell potential used for hCEC culture, they can be obtained from adipose tissue, umbilical-cord blood, or bone marrow [117,118], while cell lines can be created by inducing direct differentiation of embryonic stem cells [119], induced pluripotent stem-cells [120], and hCEC precursors [121]. Subsequently, it is essential to perform proper isolation of cells and cultivate them in a suitable culture medium supplemented with specific growth factors. Moreover, preventing endothelial-to-mesenchymal transition of hCECs during culture is crucial. Additionally, creating surfaces that mimic the native extracellular matrix environment is imperative for promoting hCEC growth and proliferation. [91].

Theorized for the first time at the beginning of the century [122], direct injection of hCEC into the anterior chamber entirely avoids the need for any carrier. The first experimental models on rabbit corneas achieved good results, as long as the post-operative prone position was maintained [123,124]. The first case of a human patient treated with this technique was reported in 2017 [125], and showed clinical improvements with corneal oedema resolution and BCVA going from 0.04 to 1.0 (decimal visual acuity). The results from the first clinical trial in humans were published later, in 2018 [126]: 11 patients were injected with a suspension of 106 cells into the anterior chamber and then placed in a prone position for 3 h, to allow the sedimentation of the injected hCEC onto the posterior surface of the cornea. Twenty-four weeks after injection, improvements in ECD, corneal thickness, and BCVA were noted. Two years after injection, corneal thickness was less than 600 µm in 10 out of the 11 eyes, while each of the 11 eyes retained corneal transparency and no immune responses were observed.

## 5. Conjunctiva 

### Mucous Membrane Pemphigoid

**Figure 6:**
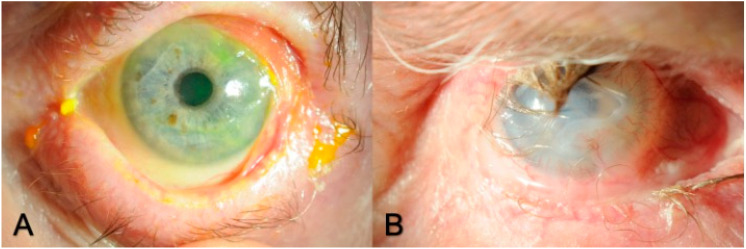
(**A**) Slit lamp photo showing active ocular mucous membrane pemphigoid: ocular inflammation, shortening of the fornix, and symblepharon; and (**B**) advanced ocular mucous membrane pemphigoid with severe ocular-surface neovascularization, corneal ulcer, and trichiasis.

Ocular mucus membrane pemphigoid (OcMMP) is a rare autoimmune cicatrizing inflammatory disease that affects the conjunctival mucosa, and it is characterized by scarring of the ocular surface with significant visual impairment (Figure 6) [127]. Frequently, also other mucosal tissues are involved, such as mouth, trachea, esophagus, larynx, and genitalia [128]. Regarding the epidemiology, OcMMP represents the first cause of cicatrizing conjunctivitis in developed countries with an incidence of about 0.8 cases per 1,000,000 per year [129]. No predisposing factors are recognized, apart from specific human leukocyte antigen (HLA): HLA-DR2 and HLA-DQw7 [130]. The median age of presentation is 65 years old [131]. Regarding the pathogenesis, the mechanism has not been fully elucidated yet. It is known that in OcMMP there is a type 2 autoimmune hypersensitivity reaction against the basal membrane of the conjunctiva triggered by systemic circulating autoantibodies which bind the antigens expressed by conjunctival epithelium basement membrane zone (BMZ) [128,132]. Clinically, the presentation may vary from non-resolving chronic conjunctivitis with symblepharon to acute conjunctivitis and limbal inflammation which may rapidly cause symblepharon [132]. Various staging systems have been proposed, among which the most used are those of Mondino and Brown [133] and Foster [127]. Mondino and Brown suggested to grade the severity of OcMMP accordingly to the degree of shortening of the inferior fornix: in Stage 1 there is a loss of depth of inferior fornix between 0 and 25%; in Stage 2 between 25 and 50%; in Stage 3 between 50 and 75%; and in Stage 4 between 75 and 100% [133]. In contrast, Foster divided the OcMMP in four stages according to the degree of scarring and fibrosis of the bulbar conjunctiva: Stage 1 if there is conjunctival scarring and fibrosis; Stage 2, in which there is a shortening of the inferior fornix; Stage 3 if there is symblepharon; and Stage 4 if the patient develops ankyloblepharon [127]. The diagnosis is achieved by a perilesional conjunctival biopsy with direct immunofluorescence (DIF) to detect immunoglobulin (IgA, IgG, and/or IgM) and/or complement (C3) deposits on the BMZ [134]. In the case of negative DIF at conjunctival biopsy, with high suspicion of OcMMP, DIF of buccal biopsy has been reported as a valid alternative [135]. The aim of the management is to slow the progression of the disease, which can lead at end stages to fully keratinized cornea. A multi-step approach management is traditionally preferred, starting with Dapsone or sulphapyridine/sulphasalazine in the case of glucose-6-phosphate dehydrogenase [136]. In moderate disease, the preferred treatment is mofetil mycophenolate (MMF), 1 gr once or twice daily (azathioprine or methotrexate in case of intolerance to MMF) [130,137]. In patients with severe OcMMP, a therapeutic option is oral or intravenous cyclophosphamide [130,137]. Other possible therapies in severe cases are represented by anti-tumor necrosis factor agents (anti-TNF) etanercept and infliximab, anti-CD-20 (rituximab), or agonist of interleukin (IL)-2 daclizumab [130]. The use of systemic steroids is useful in the short term to reach a fast control, whereas the use of long-term steroids at low dose is not beneficial [130,131,137]. Topical steroids can be used in short term and are not a substitute for systemic treatment [130].

## 6. Gene Therapies

Gene therapy is an emerging field that aims to treat ocular-surface diseases by delivering genes directly to the ocular-surface cells. The goal is to address underlying genetic defects and promote tissue regeneration. Due to its accessibility and immune privilege, the ocular surface, and, in particular, the cornea, makes it a desirable target for gene therapy [138,139,140,141,142]. Several methods of delivering genes, including viral and non-viral vectors, CRISPR-Cas9 gene editing, antisense, and siRNA therapies for epigenetic regulation, have been described in ocular-surface diseases. Although some human studies are currently available, the majority of the published results came from in vitro or animal model studies. In this paragraph, the advancements and potential uses of gene therapy in ocular-surface diseases are discussed. The process of corneal epithelial wound healing involves various growth factors, cytokines, and cell-signaling events. Gene therapy has been used to deliver target genes in diabetic corneas with slow epithelial wound healing [141,143]. Overexpression of c-Met, a gene involved in wound-healing-related processes, has been found to normalize diabetic markers and stimulate wound healing [144]. Silencing proteinases cathepsin F and MMP10 also improved diabetic epithelial wound healing [145]. Non-viral approaches, such as nanobioconjugates, have shown promise in treating diabetic corneal wound healing [146]. The anti-apoptotic gene bcl-xL has been identified as a possible target for improving corneal epithelial wound healing [147]. MiRs can also be used for gene silencing, but careful validation is necessary due to their effects on multiple targets [6,148]. Corneal injury can lead to the formation of scars and fibrosis due to the abnormal deposition of extracellular matrix proteins and the emergence of myofibroblasts [6]. TGF-β plays a vital role in this process and is a primary target of gene therapy for the prevention and treatment of fibrotic ocular-surface disease [149]. One promising approach is the use of decorin, which can form a complex with TGF-β, leading to decreased bioavailability and blocking the binding to receptors [150,151]. Studies have shown that the transfection of the decorin gene can significantly decrease the transdifferentiation of corneal fibroblasts to myofibroblasts, reducing fibrosis [152,153]. Non-viral gene therapy targeting downstream targets of TGF-β, such as soluble TGF-β receptor 2, has also shown promise in reducing myofibroblast transformation without significant cell death [154]. Wang et al. found that overexpressing the Smad7 gene using lentivirus can reduce TGF-β signaling activation in rat corneas by decreasing the phosphorylation of Smad2 and the expression of TGF-β2 after PRK surgery [155]. Additionally, in a rabbit model of corneal fibrosis induced by PRK, AAV-5-mediated Smad7 gene therapy was safely effective in inhibiting corneal scarring [156]. Gene-therapy approaches have been developed to target the angiogenic VEGF pathway and other proangiogenic factors via gene silencing or transgenic expression of antiangiogenic factors in the treatment of corneal neovascularization (CoNV) [157]. These approaches have successfully inhibited CoNV in animal models, with some clinical success achieved using antisense oligonucleotides (AON) eye drops [158]. Other promising targets for gene therapy include miR-204, anti-angiogenic pigment epithelium-derived factor, and inhibitors of proangiogenic MMP-9 and SDF-1 [159,160,161,162]. Overall, gene therapy may lead to new approved drugs for treating pathological corneal neovascularization in the future. Gene therapy has shown promising results in treating dry-eye disease in animal models. The transfer of TNF-α inhibitor gene and IL-10 gene using Ad and AAV vectors, respectively, restored tear production, reduced corneal defects, and suppressed lacrimal gland immunopathology [163]. In addition, gene therapy using AAV vectors to administer aquaporin-1 gene and MUC5AC gene has also shown improvements in dry-eye symptoms in animal models [164,165]. These findings suggest that gene therapy may hold potential for treating dry-eye disease in humans and further clinical trials are needed to test its efficacy. In conclusion, gene therapy is a promising approach for the treatment of ocular-surface diseases. Further research is needed to optimize gene-therapy vectors, delivery methods, and safety profiles to ensure these treatments’ long-term efficacy and safety. Nevertheless, the potential benefits of gene therapy in restoring visual function and improving quality of life in patients with ocular-surface diseases are substantial.

## 7. Conclusions

Regenerative therapies for ocular-surface diseases represent a promising field of research that may allow clinicians to restore the ocular-surface integrity without the need for surgery. Further, the possibility to use injectable therapies, such as endothelial cells and gene therapies, could reduce the use of corneal grafts that represent a current limit for the treatment of patients with corneal diseases.

## Figures and Tables

**Table 1:** Regenerative therapies overview for the corneal ocular-surface diseases.

Disease	Therapies
Dry-eye disease	Autologous serum tears Insulin-like growth factor Recombinant human nerve growth factor (rh-NGF)
Neurotrophic keratopathy	Recombinant human nerve growth factor (rh-NGF) Amniotic membrane Conjunctival flap Corneal neurotization
Neuropathic corneal pain	Autologous serum tears Contact lens amniotic membrane
Pterygium	Conjunctival autologous graft Amniotic membrane Antimetabolites: - mitomycin - 5-fluorouracile - anti-vascular endothelial-growth factor - cyclosporine
Limbal stem-cell deficiency	Autologous or allogenic limbal-cell transplant Cultivated limbal epithelial transplantation (CLET) Simple limbal epithelial transplantation (SLET)
Fuchs dystrophy	Descemetorhexis without endothelial keratoplasty (DWEK) RHO-kinase inhibitors (ROCK-I)

## Data Availability

Not applicable.
